# Opening Editorial: Conversations in health care education

**DOI:** 10.15694/mep.2019.000071.1

**Published:** 2019-04-01

**Authors:** Stuart Marshall, Debra Kiegaldie

**Affiliations:** 1 Monash University; 2 Holmesglen Institute

**Keywords:** Clinical Skills, Communication, Debriefing, Conversations, Clinical Education, Professionalism, Health Professions, Education

## Abstract

This article was migrated. The article was marked as recommended.

Clinical education happens predominantly via conversations. Conversations between the educator and learner, among learners, between those giving and receiving health care and even among educators striving to improve in their teaching. Sometimes these conversations may be difficult, with troublesome feedback and hard lessons to learn, sometimes they are unstructured and messy. Almost always, more dialogue is needed.

This theme coincides with the 8 ^th^ International Clinical Skills Conference in Prato, Italy. The conference has a history of starting conversations among researchers, educators and clinicians and the theme for the conference coincides with the theme for this issue. This pairing of a conference with a post publication peer review journal has not to our knowledge been previously attempted. This format gives a unique opportunity to provide a deeper learning from the peer review process. High-quality submissions will be published on the site and presented at the conference. Readers and conference attendees alike will be able to comment on the papers presented. In this way, discussions in person, online and via the traditional publishing route can be brought together to improve the publishing and research process. Indeed, the theme of ‘conversations’ could not be more apt than in these ‘metaconversations’ about educational research. We believe that the conversations within the walls and courtyard of the *Palazzo Vaj* that have influenced so many educational researchers in the past will have a chance to reach a wider audience than just the conference attendees.

Some of the types of conversations in clinical skills we will discuss have great importance in the future of clinical education and for developing health professionals with the skills needed for person-centre care. We especially wish to promote articles that highlight work undertaken in Low-Middle Income Countries (LMICs) as these align with the values of the charity that underpins the conference, the International Clinical Skills Foundation (Inc.).

## Conversations in health care education

“.. *Whatever words we utter should be chosen with care for people will hear them and be influenced by them for good or ill..*” Buddha.

## Introduction

Clinical education happens predominantly via conversations. Conversations between the educator and learner, among learners, between those giving and receiving health care and even among educators striving to improve in their teaching. Sometimes these conversations may be difficult, with troublesome feedback and hard lessons to learn, sometimes they are unstructured and messy. Almost always, more dialogue is needed.

This theme coincides with the 8 ^th^ International Clinical Skills Conference in Prato, Italy. The conference has a history of starting conversations among researchers, educators and clinicians and the theme for the conference coincides with the theme for this issue. This pairing of a conference with a post publication peer review journal has not to our knowledge been previously attempted. This format gives a unique opportunity to provide a deeper learning from the peer review process. High-quality submissions will be published on the site and presented at the conference. Readers and conference attendees alike will be able to comment on the papers presented. In this way, discussions in person, online and via the traditional publishing route can be brought together to improve the publishing and research process. Indeed, the theme of ‘conversations’ could not be more apt than in these ‘metaconversations’ about educational research. We believe that the conversations within the walls and courtyard of the *Palazzo Vaj* that have influenced so many educational researchers in the past will have a chance to reach a wider audience than just the conference attendees.

Some of the types of conversations in clinical skills we will discuss have great importance in the future of clinical education and for developing health professionals with the skills needed for person-centre care. We especially wish to promote articles that highlight work undertaken in Low-Middle Income Countries (LMICs) as these align with the values of the charity that underpins the conference, the International Clinical Skills Foundation (Inc.).

## Foundational clinical conversations

It goes without saying that preparing health professionals for core communication skills is an essential component of any pre-registration curriculum. With a third of patient complaints involving communication and interactions ( Reader, Gillepsie and Roberts, 2014) and many communication failures leading to adverse events ( Kohn, Corrigan and Donaldson, 1999; Kennedy, 2001; Thompson and V., 2009), it is critical that education providers devote time and best practice approaches to facilitating the development of these important skills. Much progress has been made in recent years to guide educators on how best to identify core communication skills, embed these into teaching and learning experiences and evaluate outcomes. The updated UK consensus statement on the content of communication curricula in undergraduate medicine including the Curriculum Communication Wheel ( Noble et al., 2018) and the European consensus on learning objectives for a core communication curriculum in health care professions ( Bachmann et al., 2013) are just two examples that could be used. In a systematic literature review and qualitative synthesis for communication skills ( Denniston et al., 2017) identified a list of 205 communication skills learning outcomes. It is clearly a complex, multi-faceted area but one that requires us to take a planned and holistic curriculum approach.

## Difficult clinical conversations

Some clinical conversations are without doubt more challenging than others because they trigger emotions, involve discussion about taboo subjects or involve subjects where language is inadequate, poorly understood or must be chosen carefully.

The role of emotions in clinical skills, and in particular communication, are perhaps the most difficult aspect of teaching and learning for health professionals. Emotions affect our cognitive processing and change how clinicians frame clinical problems. One of our keynote speakers at the conference will be Vicki LeBlanc, whose research has examined these issues. Incorporating the right levels of stress, positive and negative emotions can affect learning and the conversations we have about challenging situations in order to learn from them ( LeBlanc, 2014).

The avoidance of conflict can also lead to challenges in conversations. Liz Crowe, author, paediatric intensive care social worker and researcher will also examine how conflict and difficult emotions can take us towards important, healing conversations in her keynote address. By embracing discomfort and conflict, Liz will explain how positive outcomes can emerge.

As an international conference we also recognise that the delivery of health care to diverse cultural and social groups creates its own challenges. We especially welcome papers that address these aspects, particularly those related to challenges in Low-Middle Income Countries (LMICs).

## Learning about conversations from real and simulated patients

There is an increasing emphasis on encouraging the active participation of patients in health care and education ( Jha et al., 2009, Repper and Breeze, 2007) and the rate of publications on this topic has increased exponentially over the last 20 years. Involvement of patients in health care education is partly driven by the ethical imperative to be far more inclusive of patients as partners in their care ( Towle et al., 2016). Much of the evidence in this field refers to the benefits this brings to patients, learners, health care organisations and communities. Despite the strong impetus for a patient focus there is little written about effective models of education and the strategies that work when integrating patients into teaching and learning experiences and how we can learn from patients. As patients are the primary recipient of clinical conversations they have the potential to play a significant role in communication skills education. The drive towards inclusion of the patient’s voice in health and social care education is underscored by the 2016 Vancouver Statement that highlights the future direction and priorities for action for patient involvement in clinical education ( Towle et al., 2016).

In his keynote address and workshop, Jimmie Leppink will investigate how we talk about objective and subjective measures of assessment to model and understand the learning process. A conversation that includes patient measures as well as more traditional metrics of examination and summative assessment is something that will be considered.

The role of the simulated participant in health care education is equally important. They provide the physical and pscychological embodiment of the real patient and as such deliver crucial contributions to the education of health professionals, particularly in relation to conversational feedback ( Nestel, Roche and Battista, 2017).

## Professional conversations

Inter- and intraprofessional conversations are also learning conversations. Interprofessional conversations are those that take place between health professionals and intraprofessional conversations take place within professions. Whilst interprofessional learning has received much attention over recent decades, very little research has been done on fostering collaboration and consultation within the professions. All are necessary conversations that require greater emphasis and integration into our health care education to promote respect, teamwork, collaboration and person-centred care.

Another two of our keynote speakers, Tanja Manser and Walter Eppich will address how formal and informal conversations in both simulation and clinical work can best develop clinical skills. Furthermore, the reverse conversation and associated tools such as the PEARLS debriefing feedback system can inform the practice of the educator and provide feedback to improve the effectiveness of the educator ( Eppich and Cheng, 2015).

Finally, the International Clinical Skills Conference is a conference that embraces social media conversations. Twitter, Facebook, Instagram and other platforms are becoming essential tools to augment education in the clinical and classroom setting. We welcome papers that describe how these new technologies can connect students, educators and researchers from around the world.

## Take Home Messages

Conversations are the lifeblood of education. The ability to explain and explore these conversations in contemporary health professional education is central to our capability of providing a health workforce with the compassion and skills required for safe, effective and humanistic care. We look forward to the emerging conversations about this research here and the potential that a full open discussion on these topics can bring.

## Notes On Contributors

Stuart Marshall is an anaesthesiologist, clinical educator and researcher in human factors / ergonomics at Monash University in Melbourne. He is also an Honorary Clinical Associate Professor in Medical Education at the University of Melbourne. ORCID: https://orcid.org/0000-0002-5406-9279

Debra Kiegaldie is the clinical chair of healthcare and community simulation at the Holmesglen Institute in Melbourne, Australia. She is also an Honorary Associate Professor at the Eastern Clinical School, Monash University. ORCID: https://orcid.org/0000-0002-4077-5818

## Declarations

Debra Kiegaldie and Stuart Marshall are guest theme editors for the “Conversations about Clinical Skills” themed issue in AMEE MedEdPublish. Stuart Marshall is the Chair of the International Clinical Skills Conference committee and International Clinical Skills Foundation (Inc.)
