# Supplementary material for: Opening Editorial: Conversations in health care education
Source: MedEdPublish (2016). 2019 Apr 1;8:71. [Version 1] doi: 10.15694/mep.2019.000071.1 (PMC10712593; doi:10.15694/mep.2019.000071.1)
Supplement: Peer review history

# Reviewer report: Reviewer response for version 1

**DOI:** 10.21956/mep.19821.r29856

**Published:** 2019-04-25

**Authors:** Richard Hays

**Affiliations:** 1 James Cook University

This review has been migrated. The reviewer awarded 5 stars out of 5 I would like to thank the authors for this opening editorial, which outlines the current state in academic clinical skills development and some of the questions yet to be addressed. MedEdPublish is planning to work with the convenors of the International Clinical Skills Conference to facilitate conversations about research within the clinical skills theme, some of which are likely to be published during this issue of MEP.

Reviewer Expertise:

NA

No decision status is available

# Reviewer report: Reviewer response for version 1

**DOI:** 10.21956/mep.19821.r29857

**Published:** 2019-04-05

**Authors:** Megan Anakin

**Affiliations:** 1 University of Otago

This review has been migrated. The reviewer awarded 5 stars out of 5 Thank you for writing this editorial to promote the link between this themed edition of MedEdPublish with an upcoming conference. The authors have identified several research and practice gaps involving clinical skills, and professional communication in particular. I look forward to reading about how these conversations develop at the conference, in social media, and later in MedEdPublish.

Reviewer Expertise:

NA

No decision status is available
